# Dexamethasone Sensitizes Acute Monocytic Leukemia Cells to Ara-C by Upregulating FKBP51

**DOI:** 10.3389/fonc.2022.888695

**Published:** 2022-07-04

**Authors:** Huanxin Sun, Xiaowen Liu, Laicheng Wang, Bin Cui, Wenli Mu, Yu Xia, Shuang Liu, Xin Liu, Yulian Jiao, Yueran Zhao

**Affiliations:** ^1^ Department of Central Laboratory, Shandong Provincial Hospital, Shandong University, Jinan, China; ^2^ Center for Reproductive Medicine, National Research Center for Assisted Reproductive Technology and Reproductive Genetics, The Key Laboratory for Reproductive Endocrinology of Ministry of Education, Shandong University, Jinan, China

**Keywords:** acute monocyte leukemia (AML-M5), dexamethasone (DEX), FK506 binding protein 51 (FKBP51), serine/threonine protein kinase (AKT), cytarabine (Ara-C), glycogen synthase kinase-3 (GSK3β), forkhead box O1A (FOXO1A)

## Abstract

In this study, we demonstrated that the expression of FK506 binding protein 51 (FKBP51) is upregulated in acute monocytic leukemia (AML-M5) cells by dexamethasone and aimed to investigate the possible effects of FKBP51 on the growth and cytarabine sensitivity of AML-M5 cells. THP-1 and U937cells were used to establish AML-M5 cell models with FKBP51 overexpression and knockdown, respectively. Cell proliferation, apoptosis and response to cytarabine were investigated by cell cycle, CCK-8 and Flow cytometry analyses. The mice experiment was conducted to detect the role of FKBP51 on AML-M5 cells proliferation and antileukemia effect of Ara-C/Dexamethasone co-therapy *in vivo*. Western blots were employed to determine protein expression levels. FKBP51 upregulation significantly attenuated THP-1 cell proliferation and sensitized the cells to cytarabine treatment which was further enhanced by dexamethasone. These effects were indicated by decreases in cell viability, S-G2/M phase cell cycle distribution, cytarabine 50% inhibitory concentration (IC50) values and increases in apoptosis and were supported by decreased phosphorylation levels of AKT, GSK3β and FOXO1A and decreased levels of BCL-2 and increased levels of P21 and P27. In contrast, FKBP51 knockdown led to excessive U937 cell proliferation and cytarabine resistance, as indicated by increased cell viability and S-G2/M phase cell cycle distribution, decreased apoptosis, increased phosphorylation levels of AKT, GSK3β and FOXO1A, and increased BCL-2 and decreased P21 and P27 expression. In addition, an AKT inhibitor blocked cell cycle progression and reduced cell viability in all groups of cells. Furthermore, SAFit2, a specific FKBP51 inhibitor, increased U937 cell viability and cytarabine resistance as well as AKT phosphorylation. In conclusion, FKBP51 decelerates proliferation and improves the cytarabine sensitivity of AML-M5 cells by inhibiting AKT pathways, and dexamethasone in combination with Ara-C improves the chemosensitivity of AML-M5.

## Introduction

Acute myeloid leukemia (AML) is a group of heterogeneous haematologic malignancies that can result from genetic mutations or epigenetic modifications that occur in many different combinations (1). Abnormal proliferation and differentiation cause elevated immature myeloid cell numbers and loss of function (2). Among the AML subtypes, acute monocytic leukemia (AML-M5) is a common subtype in adults. Ara-C, a pyrimidine analogue that inhibits DNA replication, is the most effective drug against AML (3). However, the progression and patient response to chemotherapy in AML-M5 vary greatly (4). Despite the initial achievement of complete remission with standard induction agents in 60–80% of younger patients with AML, only ∼ 30% are alive and disease free at 5 years. Resistance to chemotherapy is a major obstacle to successful outcomes for many patients with AML (5). Hence, elucidating the mechanisms underlying abnormal proliferation and drug resistance in AML-M5 and identifying new biomarkers and therapeutic targets are urgently needed to enhance the chemotherapy efficacy.

FK506 binding protein 51 (FKBP51) is a 51-kDa protein that belongs to a family of immunophilins. Structurally, FKBP51 contains two N-terminal FK506 binding domains (FK1 and FK2) and C-terminal tetratricopeptide repeat (TPR) domains (6). The FK1 domain has peptidylprolyl cis-trans isomerization (PPIase) activity and immunosuppressant agent binding ability and is thus involved in immunoregulation, protein folding and trafficking. The FK2 domain seems to have only a structural role (6, 7). The C-terminal TPR domain binds to heat shock protein 90 (Hsp90) and assembles with all steroid receptors. In cultured human cells, FKBP51 gene expression was robustly upregulated by glucocorticoids and was suggested as a bioassay for detecting individual sensitivity to corticosteroids (8–10). Furthermore, glucocorticoids are globally being used for the treatment of chemotherapy-induced nausea (11) and acute respiratory distress syndrome (12) in patients with AML.

In addition to participating in the regulation of steroid hormones responses and hormone receptor activities, increasing evidence indicates that FKBP51 plays a role in the abnormal cell growth of cancers, and it could be considered a promising new marker of tumor progression and response to radio/chemotherapy (13, 14). This protein inhibits lung adenocarcinoma, pancreatic cancer and glioma *via* its scaffolding function, which negatively regulates AKT phosphorylation (15–17). Moreover, FKBP51 is reported to promote the progression of prostate cancer and melanoma through the NF-κB signaling pathway (18, 19).

However, the role of FKBP51 in human haematological malignancies has not been understood until now. In the present study, we aimed to investigate the role of FKBP51 in AML-M5 cell growth and cytarabine (Ara-C) chemotherapy sensitivity, as well as the underlying molecular mechanisms Meanwhile, we hypothesized that dexamethasone is likely to increase vulnerability to Ara-C.

## Materials and Methods

### Reagents

Dexamethasone (Dex) were purchased from Selleck (Selleck Chemicals, United States). The FKBP51 shRNA plasmid (h) (sc-35380-sh), scramble shRNA (sc-108060) and transfection reagent (sc-108061) as well as the primary antibodies against FKBP51 (sc-271547) and GAPDH (sc-47724) were purchased from Santa Cruz Biotechnology, Inc. (Shanghai, China). FKBP51 lentiviral expression vectors were constructed and packaged by Shanghai GeneChem BioTECH (Shanghai, China). Puromycin, Ara-C and AKT inhibitor (A6730) were purchased from Sigma-Aldrich Trading Co., Ltd. (Shanghai, China). Primary antibodies against phospho-GSK3β (S9) (ab75814), GSK3β (ab32391), P21 (ab109520), P27 (ab32034), p-FOXO1A (S256) (ab31339), FOXO1A (ab52857), BAX (ab32503) and BCL-2 (ab32124) were purchased from Abcam (Shanghai, China). The primary antibodies against phospho-AKT (Ser473) (4060S) and AKT (pan)(4685S) were purchased from Cell Signalling Technology, Inc. (Danvers, MA, USA). The goat anti-rabbit horseradish peroxidase-labelled secondary antibody (Z2301) was purchased from Beijing Zhongshan Jinqiao Biotechnology Co., Ltd. (Beijing, China).

### Cell Lines and Culture

U937 and THP-1 cells were purchased from the Chinese Academy of Science (Shanghai, China). U937 cells were grown in Roswell Park Memorial Institute (RPMI) 1640 basic medium (C11875500CP, Gibco^®^ Thermo Fisher Scientific, Suzhou, China) supplemented with 10% fetal bovine serum (FBS) (Gibco, Suzhou, China) and 1% penicillin/streptomycin at 37°C in a 5% CO2 humidified atmosphere. THP-1 cells were grown in specific medium for THP-1 cells (CM-0233, Procell Life Science & Technology Co., Ltd., Wuhan, China) at 37°C in a 5% CO2 humidified atmosphere.

### Lentivirus Infection

The THP-1 cells (5×10e5) were seeded in 24-well tissue culture plates in antibiotic-free medium supplemented with 10% FBS and cultured overnight. The cells were infected with FKBP51 lentivirus and an empty lentiviral vector for 8 hours at a multiplicity of infection (MOI) of 50. The empty lentiviral vector was used as a negative control. Subsequently, the medium was replaced with fresh complete medium. The percentage of lentivirus-infected cells, which were green fluorescent protein (GFP)-positive, was determined with a fluorescence microscope 72 hours after infection (Molecular Devices IamgeXpress^®^ Micro Confocal System, San Jose, CA, USA).

### FKBP51 shRNA Plasmid Transfection

U937 cells (5×10e5) were seeded in 24-well tissue culture plates with antibiotic-free RPMI 1640 medium supplemented with 10% FBS overnight. Next, 0.2 μg of shRNA and negative control plasmids were transfected into U937 cells with 3 µl of the corresponding transfection reagent according to the manufacturer’s instructions. The cells were infected for 8 hours, and the medium was then replaced with fresh complete medium. After 2 days, these transfected cells were screened with 2 µg/ml puromycin in RPMI 1640 medium supplemented with 10% FBS. The positive transfectants were obtained and expanded.

### RNA Isolation and qPCR

Total RNA was extracted using a MiniBEST Universal RNA Extraction Kit (TaKaRa, Tokyo, Japan). Then, RNA (1 μg) was reverse transcribed using the Transcriptor First-strand cDNA Synthesis Kit (Roche Applied Science, Mannheim, Germany) and random hexamer primers in a final volume of 20 μl. qPCR was performed using a Roche LightCycler 480 and SYBR green (TaKaRa Bio Inc, Dalian, China) to quantify FKBP51 mRNA levels under the following conditions: 10 min at 95°C; 45 cycles of 95°C for 10 sec, 60°C for 10 sec; and 72°C for 10 sec. GAPDH was used as a control to compare gene expression. The specific primer pairs were designed as follows: FKBP51: 5’-AAA AGG CCA AGG AGC ACA AC-3’ (sense), 5’-TTG AGG AGG GGC CGA GTT C-3’ (antisense); GAPDH: 5’-GGT ATC GTG GAA GGA CTC-3’ (sense), and 5’-GTA GAG GCA GGG ATG ATG-3’ (antisense). The ΔΔCt method was used to calculate the relative expression of the gene (20).

### Western Blotting

Cellular protein was extracted using a cell protein extraction kit (Thermo Fisher Scientific, Waltham, MA, USA), and protein concentrations were determined using the Pierce BCA protein assay kit (Thermo Fisher Scientific, Waltham, MA, USA). In total, 20 µg of protein per lane was separated by sodium dodecyl sulfate polyacrylamide gel electrophoresis (SDS-PAGE) on 10% separating gels, and the proteins were then transferred onto polyvinylidene fluoride (PVDF) membranes. Following the transfer, the membranes were blocked with 5% non-fat dried milk in Tris buffered saline Tween (TBST) for 1 hour at room temperature and incubated overnight at 4°C with primary antibodies (the dilution of the antibodies against p-GSK3β and GSK3β was 1:5000, and the dilution for the other primary antibodies was 1:1000). The PVDF membranes were washed 3 times with TBST and then incubated with horseradish peroxidase-labelled secondary antibody (1:5000 dilution) for 1 hour at room temperature. After washing 3 times with TBST, the immunoreactive bands were visualized using ImmobilonTM Western HRP Substrate Peroxide Solution (WBKLS00100, Millipore, Billerica, MA, USA). GAPDH was used as an internal control for total protein. Each experiment was performed in triplicate. Image-Pro Plus (version 6.0.0.260, Media Cybernetics, Inc. Maryland, America) was used to quantify the protein bands.

### Cell Proliferation Assay

Cells were plated in triplicate in 96-well plates at a density of 5×10e3 cells per well, and CCK-8 assays were performed at the indicated time points. The optical density of each well was measured at 450 nm using a Bio-Tek microplate reader (Bio-Tek Instruments, Thermo Fisher Scientific, Winooski, VT). Triplicate plates were used for each time point, and each data point is the average of three experiments.

### Apoptosis Assay

Cell apoptosis was measured by the PE Annexin V apoptosis detection kit (BD Bioscience, San Jose). The cells of each group cultured in 24-well plates were collected and washed twice with PBS and then resuspended in binding buffer. Then, 5 µL of PE Annexin V and 5 µL 7AAD were added. The mixture was incubated for 15 minutes at room temperature in the dark. The total numbers of AnnexinV+ 7AAD− apoptotic cells were determined in a flow cytometer (Becton Dickinson, Franklin Lakes, NJ, USA).

### Cell Cycle Analysis

A total of 1×10e6 cells were collected and washed with PBS before being fixed in 70% ice-cold ethanol. After incubation for at least 3 hours at -20°C, the cells were washed again and incubated with 200 μl of Muse™ Cell Cycle Reagent (Millipore, Billerica, MA, USA. cat # MCH100106) containing propidium iodide (PI) for 30 min at room temperature in the dark. The percentages of cells in the G0/G1, S and G2/M phases were read on a Muse Cell Analyzer (Millipore, Billerica, MA, USA). Each experiment was performed in triplicate, and the data are expressed as the mean ± SD.

### *In Vivo* Xenograft Mice Model

Male NOD.CB17-Prkdcscid/NcrCrl (NOD/SCID) mice (5 weeks old) were purchased from Beijing Vital River Laboratory Animal Technology Co., Ltd. (Beijing, China). To investigate the effect of FKBP51 on tumor proliferation *in vivo*, a number of 1x10e7 THP-1 FKBP51-OE or THP-NC stably transfected cells suspended in 100 µl of medium were injected into right forelimb of each mouse subcutaneously (n=5 per group). The primary tumor size was measured from the outside of the mouse’s skin every 2 days and calculated by the following formula: volume=1/2 × length × width^2^. After 3 weeks, tumor nodules were surgically excised for further analysis. In addition, the ectopic tumors were further induced to detect the antileukemia effect of Ara-C/Dex co-therapy *in vivo*. THP-1 cells were inoculated subcutaneously, and then all mice with successful tumor-forming were divided into four groups including the vehicle group, Ara-C group (30 mg/kg/day), Dex group (5 mg/kg/day) and Ara-C (30 mg/kg/day)/Dex (5 mg/kg/day) combination group. When tumors had a tactile sensation, each group began to receive medication using intraperitoneal injection (vehicle group used saline). After 10 days of drug treatment, the study was terminated. The animal studies were approved by the Medical Ethics Committee of Shandong Provincial Hospital.

### Statistical Analysis

The data were analyzed with GraphPad Prism 8.0 (CA, USA). Student’s t test or one-way ANOVA were used to compare data. p < 0.05 was considered statistically significant.

### Results

#### FKBP51 Suppresses AML-M5 Cells Proliferation *In Vitro* and *In Vivo*

**Figure 1:**
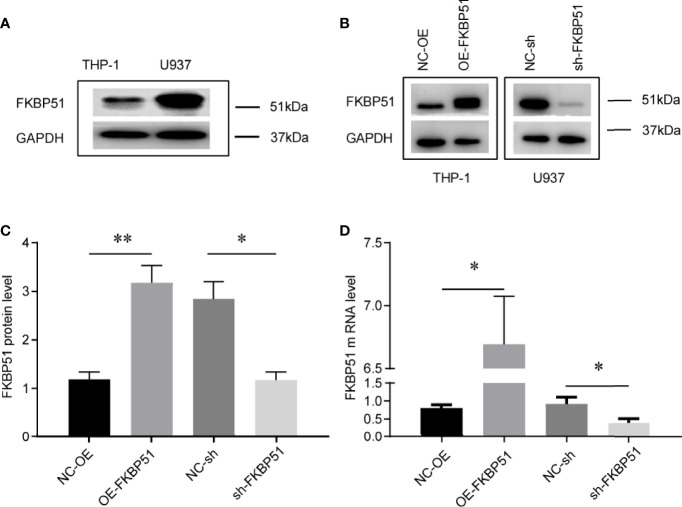
Cell models of FKBP51 overexpression and knockdown were constructed and verified. **(A)** Relative expression of FKBP51 protein in U937 cells and THP-1 cells, which was confirmed by western blot analysis. **(B-D)** The expression of FKBP51 protein **(B, C)** and mRNA **(D)** were increased in FKBP51 over-expressed THP-1 cells and decreased in FKBP51-knockdown U937 cells. GAPDH was used as internal control. Each experiment was performed in triplicate. OE-FKBP51, FKBP51-overexpressing THP-1 cells; NC-OE, negative control for OE-FKBP51; sh-FKBP51, shRNA-FKBP51-treated U937 cells; NC-sh, negative control for sh-FKBP51. The data are shown as the mean ± SD, and differences between groups were analyzed using independent sample Student’s t test and considered to be significant at p < 0.05 (* for p < 0.05, ** for p < 0.01).

**Figure 2:**
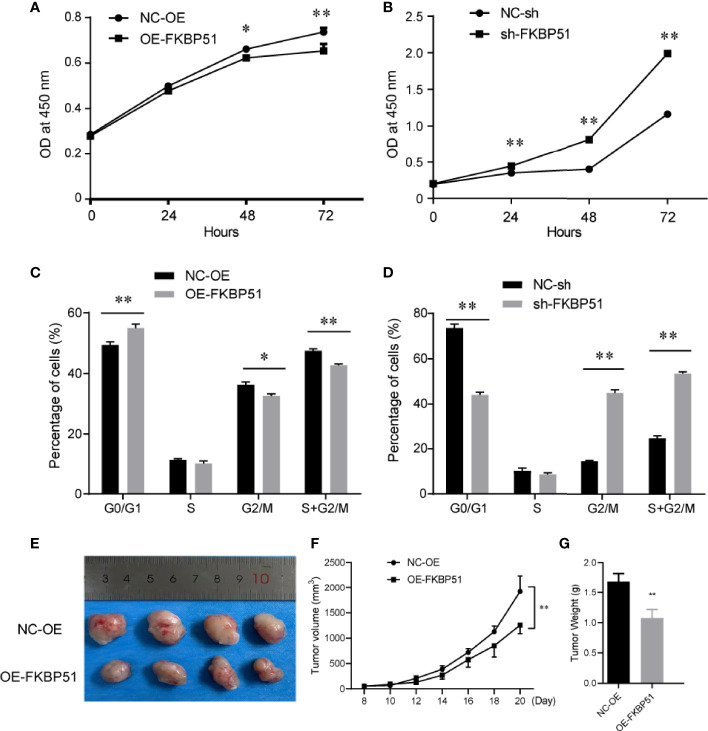
FKBP51 suppresses the cell cycle progression and proliferation of AML-M5 cells both *in vitro* and *in vivo* **(A, B)** In the CCK-8 cell proliferation assays, the upregulation of FKBP51 suppressed the growth rate of AML-M5 cells **(A)**, while downregulation had the opposite effect **(B)**. **(C, D)** Overexpression of FKBP51 resulted in a decrease in the G2/S ratio in AML-M5 cells **(C)**, while downregulation had the opposite effect **(D)**. **(E)** up-regulation of FKBP51 decreased tumor growth *in vivo*. Top, tumors from the control cells after 3 weeks of implantation; bottom, tumors from cells with stable overexpression of FKBP51. Images were photographed at the time of grafted removal (day 21). **(F, G)** The weight and volume of tumors from the FKBP51overexpression cells were significantly lower than those from the control cells (p < 0.01). All data are shown as the means ± SD. *p < 0.05, **p < 0.01.

FKBP51 function was analyzed in THP-1 and U937 cell lines. The baseline expression levels were determined by western blot, FKBP51 protein levels were markedly higher in the U937 cells and lower in THP-1 cells (**Figure 1A**). We overexpressed FKBP51 by infecting lentivirus into THP-1 and knocked down the FKBP51 expression by shRNA plasmid transfected to U937 cells. The knockdown and overexpression efficiency were evaluated by qPCR and western blot. The mRNA and protein expression of FKBP51 were increased in FKBP51 lentivirus infected THP-1 cells and decreased in FKBP51 shRNA plasmid transfected U937 cells compared with their negative control cells(**Figures 1B–D**). These results suggested that AML-M5 cell models with FKBP51 overexpression and knockdown were successfully established. To investigate if FKBP51 protein could modulate cell proliferation, cell counting kit-8 (CCK-8) assay was performed. The results indicated that overexpression of FKBP51 significantly inhibited the growth of THP-1 cells, whereas the knockdown of FKBP51 significantly accelerated the growth of U937 cells (**Figures 2A, B**). Cell cycle distribution was evaluated with Muse Cell Analyzer. As shown in **Figure 2C**, FKBP51 overexpressed THP-1 cells showed an increased number of G0/G1 phase cells and decreased S-G2/M phase cells. Conversely, FKBP51-knockdown U937 cells exhibited a decrease in the number of G0/G1 phase cells and an increase in S-G2/M phase cells (**Figure 2D**). These results suggested that overexpression of FKBP51 suppresses THP-1 cell growth by inducing cell cycle arrest at the G0/G1 phase and that FKBP51 knockdown promotes U937 cell proliferation by accelerating S-G2/M phase transformation.

After studying the effect of FKBP51 on cell proliferation *in vitro*, the involvement of FKBP51 protein in carcinogenesis *in vivo* was investigated.THP-1 cells stably transfected with FKBP51 lentivirus and their controls were subcutaneously injected into the flanks of NOD/SCID mice. As one mouse in each group failed to form tumor, the remaining four mice in each group were used for subsequent experiments. The sizes of the tumor were measured every 2 days. After 21 days, the study was terminated and tumor nodules were surgically excised for further analysis. Images were photographed at the time of grafted removal (day 21) (**Figure 2E**). The weight and volume of tumors from the FKBP51 -OE group were significantly less than those of the controls (**Figures 2E–G**). These data demonstrated that overexpression of FKBP51 could inhibit the growth of tumor *in vivo*.

#### Dexamethasone Upregulated FKBP51 Expression and Inhibited AML-M5 Cell Proliferation

**Figure 3:**
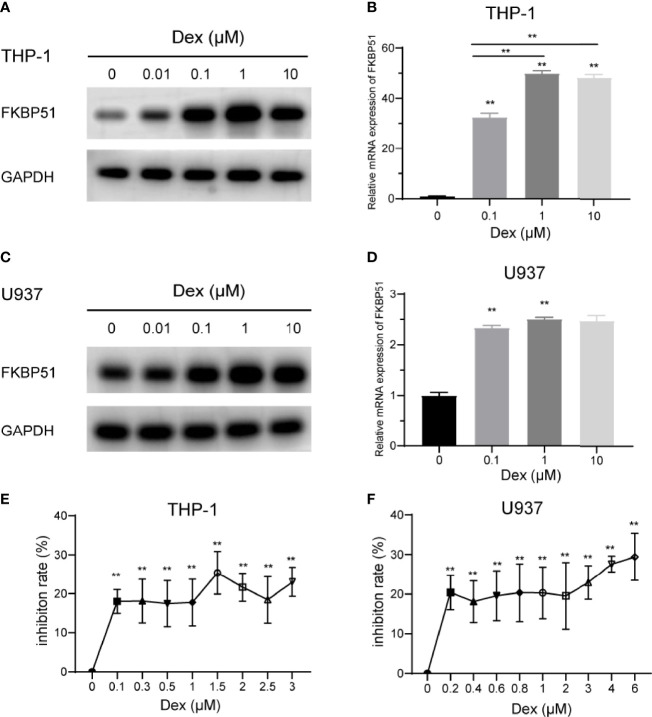
Effect of dexamethasone on the FKBP51expression and proliferation of THP-1 and U937 cells. **(A–D)** The relative protein and mRNA quantities of FKBP51 in THP-1 and U937 cells conditioned with dexamethasone at a series of concentration relative to that in control cells. Dexamethasone upregulated FKBP51 expression in a dose-dependent manner. **(E, F)** After treatment with or without dexamethasone for 48 hours, the growth of AML-M5 cells was assessed by the CCK-8 assay. Dexamethasone slightly suppressed the cell viability of both cell groups while the inhibition rate did not increase with Dex concentration. The data are shown as the mean ± SD, and differences between groups were analyzed using independent sample Student’s t test and were considered to be significant at p < 0.05 (**p < 0.01).

Both discovery and research motivation to study FKBP51 is tightly connected to the investigation of steroid hormone receptors. Here we explored the effect of Dex on FKBP51 expression and proliferation in monocytes. THP-1 and U937 cells were cultured in medium alone or with Dex at concentrations up to 10 μM, FKBP51 expression was measured by Western blot and qPCR. As shown in **Figures 3A–D**. FKBP51 was significantly upregulated after treatment with Dex at concentrations greater than or equal to 0.01 μM. As the highest expression was induced by Dex 1μM, this dose was chosen for the following experiment. Next, we explored the effect of Dex on theproliferation of THP-1 and U937 cells. Dex slightly inhibited cells proliferation. However, it is interesting to note that THP-1 and U937 cells are refractory to growth inhibition by Dex as growth inhibition did not increase with DEX concentration (**Figures 3E, F**).

#### FKBP51 Sensitizes AML-M5 Cells to Ara-C Chemotherapy

**Figure 4:**
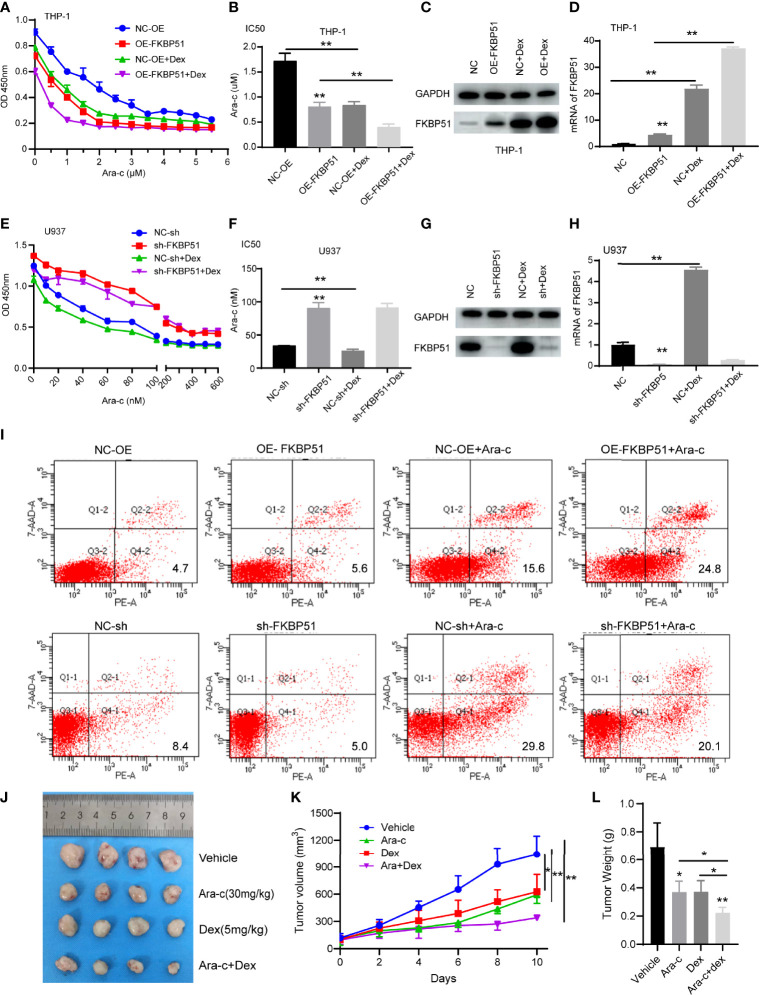
Dexamethasone enhances the sensitivity of AML-M5 cells to Ara-C by up-regulating FKBP51. **(A, E)** FKBP51 enhances the sensitivity of AML-M5 cells to Ara-C. After treatment with increasing concentrations of Ara-C with or without 1 μM dexamethasone for 48 hours, the growth of FKBP51-overexpressed THP-1 cells **(A)** and FKBP51-knockdown U937 cells **(E)** and of the corresponding negative control cells was assessed by CCK-8 assay. **(B, D)** The corresponding IC50 values for **(A, D)** are shown as the mean ± SD. **(C, D)** Western blot and qPCR experiments were applied to detect the expression level of FKBP51 in FKBP51-overexpressed THP-1 cells after Dex (1μM) treated for 24 h. **(G, H)** Western blot and qPCR experiments were applied to detect the expression level of FKBP51 in FKBP51-knockdown U937 cells and corresponding negative control cells after Dex (1μM) treated. **(I)** Percent apoptosis of control and FKBP51overexpressing THP-1 AML cells treated with or without cytarabine by FACS analysis. **(F)** Percent apoptosis of control and FKBP51 knockdown U937 cells treated with or without cytarabine by FACS analysis. **(J-L)** Dex enhanced the anti-leukemia effect of Ara-C on AML mouse xenograft model. The mice with successful tumor-forming were treated with saline (vehicle group), Ara-C (30 mg/kg/d), Dex (5 mg/kg/d), Ara-C/Dex by intraperitoneal injection. **(J)** Image of the tumors in vehicle, Ara-C, Dex and Ara- C/Dex four groups were recorded when the experiment ended. **(K)** Mean tumor volume in four groups were measured at 2 days intervals. **(L)** Weight of all tumors in four groups was measured after ending of administration Each experiment was performed in triplicate. Statistical differences from negative control cells are indicated by asterisks (* for p < 0.05, ** for p < 0.01).

Ara-C is a conventional chemotherapeutic drug for AML. After treating the cells with a series of concentrations of Ara-C, we measured the cell viability and calculated the 50% inhibitory concentration (IC50). We observed that overexpression of FKBP51 sensitized THP-1 cells to Ara-C, whereas FKBP51 knockdown in U937 increase resistance to Ara-C. The results of CCK-8 assays showed that the OD450 decreased in an Ara-C dose-dependent manner in all groups of cells (**Figures 4A, E**). The IC50 value of FKBP51 overexpressed THP-1 cells decreased and that of FKBP51-knockdown U937 cells increased compared with the values in the corresponding control cells (**Figures 4B, F**). To assess the impact of FKBP51 on apoptosis, flow cytometry experiments were performed. A 24 h exposure to the Ara-C (**Figure 4I**) markedly increased the apoptosis percentage in FKBP51 overexpressed THP-1 cells. The opposite result was observed in the FKBP51-knockdown U937 cells. These results suggested that FKBP51 overexpression sensitized AML-M5 cells to Ara-C by inhibiting proliferation and promoting apoptosis.

#### Dexamethasone Sensitizes AML-M5 Cells to Ara-C by Upregulating FKBP51

As our study showed that FKBP51overexpression enhances Ara-C sensitivity and FKBP51 can be up-regulated by Dex, so we want to know the role of Dex in AML-M5 treatment. We measured the cell viability with or without Dex when treatment with Ara-C. The results of CCK-8 assays showed that the cell viability and IC50 was further decreased in all groups except the FKBP51-knockdown U937 cells (**Figures 4A, B, E, F**). To explain this phenomenon, we measured FKBP51 expression levels by Western blot and qPCR. Consistent with the cell function experiment results, FKBP51 levels were further increased in THP OE-FKBP51cells and its control (**Figures 4C, D**). The FKBP51 expression levels were also upregulated by Dex in U937-NC group. However, the sensitizing effect of Dex on Ara-C disappeared when FKBP51 knocked down in U937 (**Figures 4G, H**). These data suggest that Dexamethasone sensitizes AML-M5 cells to Ara-C by upregulating FKBP51.

The mice experiment was conducted to detect the antileukemia effect of Ara-C/Dex co-therapy *in vivo*. The first step was the establishment of AML nude mice xenograft model. 5 × 10e6 THP-1 cells were inoculated subcutaneously, and then all mice with successful tumor-forming were divided into four groups including the vehicle group, Ara-C group (30 mg/kg/day), Dex group (5 mg/kg/day) and Ara-C (30 mg/kg/day)/Dex (5 mg/kg/day) combination group. When tumors had a tactile sensation, each group began to receive medication using intraperitoneal injection. After 10 days of drug treatment, the study was terminated and the results from the experiment were displayed (**Figure 4J**). The average tumor size in the vehicle group was 1042 ± 204.1 mm^3^, in the Dex and Ara-C groups were 627.3 ± 191.4 mm^3^ (P < 0.05) and 592 ± 47.59 mm^3^ (P < 0.05), respectively, and the Ara-C/Dex group was 340.9 ± 13.75 mm^3^ (P < 0.05) (**Figure 4K**). The average weight of tumors in the vehicle group was 0.69 ± 0.17 g, in the Dex or Ara-C groups decreased to 0.373 ± 0.08 g (P < 0.05) and 0.37 ± 0.07 g (P < 0.05), respectively (**Figure 4L**). The Ara-C/Dex group decreased the most to 0.22 ± 0.04 g (P < 0.001). These results revealed that Dex and Ara-C markedly inhibited the proliferation of AML cells, and the antiproliferative effect was more notable following Ara-C/Dex co-treatment.

#### FKBP51 Induces Inactivation of the AKT Pathway

**Figure 5:**
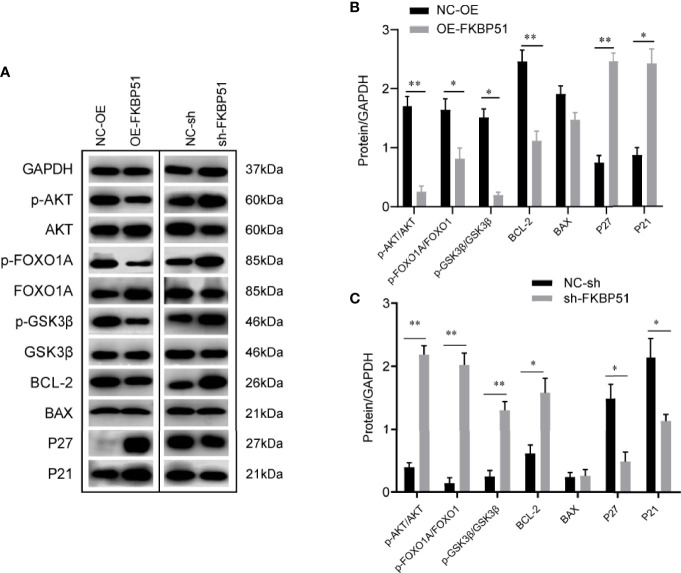
The effect of FKBP51 on the expression level of AKT pathway-related proteins. **(A)** Phosphorylated (Ser473) and total AKT and AKT downstream targets (P21, P27, BAX, BCL-2, total and phosphorylated Foxo1A, and total and phosphorylated GSK3β) in AML-M5 cells with different expression levels of FKBP51 were detected by western blot. GAPDH was used an internal control. **(B, C)** Statistical results of AKT-related protein levels in FKBP51 overexpressed THP-1 cells and FKBP51-knockdown U937 cells as well as their corresponding control cells. All cells were treated with LPS (1 μg/ml) for 15 minutes before protein extraction. Each experiment was performed in triplicate. Statistical differences from negative control cells are indicated by asterisks (* for p < 0.05, ** for p < 0.01).

AKT pathway plays important roles in cell survival and proliferation. Furthermore, several studies have explored the role of FKBP51 in AKT pathway in pancreatic cancer, endometrial adenocarcinomas, glioma (16, 21, 22). Here, we investigated the effects of the FKBP51 expression level on AKT pathway activity and its downstream target genes in AML-M5 cells. As shown in **Figure 5**, the phosphorylation of AKT on Ser473 was decreased in FKBP51 overexpressed THP-1 cells compared with that in negative control cells, and there was impaired phosphorylation of GSK3β on serine 9 (Ser9) and FOXO1A on serine 256 (Ser256), decreased BCL-2 expression and elevated P21 and P27 expression, which are related to cell cycle progression, cell proliferation and apoptosis (**Figures 5A, B**). The opposite results were observed in FKBP51-knockdown U937 cells (**Figures 5A, C**). These results implied that FKBP51 negatively regulates pSer473-AKT and its downstream target proteins.

#### The AKT Inhibitor Inhibits Cell Growth by Downregulating the Activity of the AKT Pathway

**Figure 6:**
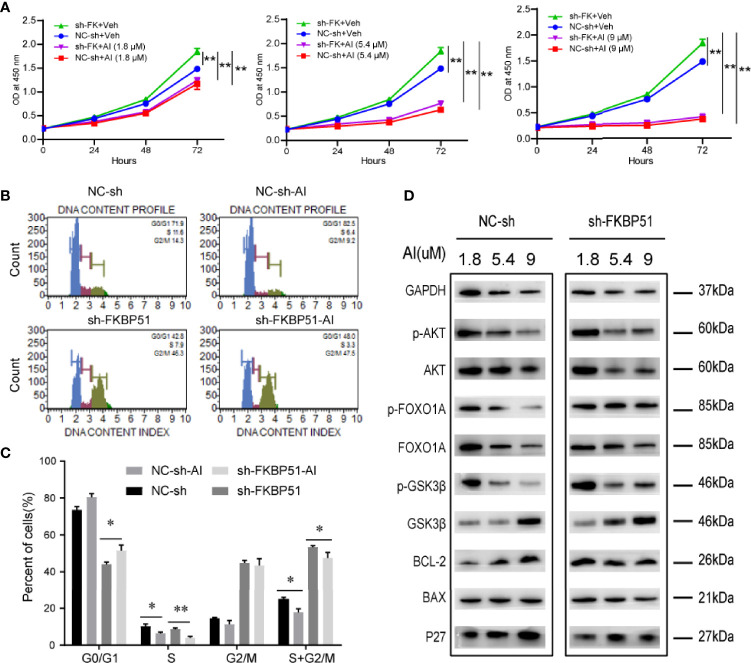
The AKT inhibitor inhibited the proliferation and cycle of AML-M5 cells. **(A)** FKBP51-knockdown U937 cells and negative control cells were treated with different concentrations of AI and a CCK-8 assay was performed. The absorbance at 450 nm of each group was used to reflect cell proliferation. **(B, C)** The effect of the AKT inhibitor on the cell cycle in FKBP51-knockdown U937 cells and control cells. **(D)** After treating FKBP51-knockdown U937 cells and negative control cells with AKT inhibitors (1.8, 5.4 and 9 μM) for 24 hours, western blot analysis of total and phosphorylated (Ser473) AKT and AKT downstream targets (p27, BAX, BCL-2, total and phosphorylated (Ser256) Foxo1, and total and phosphorylated (Ser9) GSK3β) was performed. NC-sh, negative control for sh-FKBP51; sh-FKBP51, shRNA-FKBP51; AI, AKT inhibitor. All cells were treated with LPS (1 µg/ml) for 15 min before protein extraction. Each experiment was performed in triplicate. Statistical differences from negative control cells are indicated by asterisks (* for p < 0.05, ** for p < 0.01).

To confirm the significance of AKT activity in the growth of AML-M5 cells, we treated the cells with an AKT inhibitor (AI) (A6730) and analyzed the cell proliferation and cycle. The A6730 abolished FKBP51-shRNA induced cell proliferation (**Figure 6A**). The number of G0/G1 phase cells increased, and the number of S-G2/M phase cells decreased (5.4 µM) (**Figures 6B, C**); At the protein level, FKBP51-knockdown U937 cells and control cells showed AI dose-dependent (1.8, 5.4 and 9 µM) decreases in pSer473-AKT, pSer9-GSK3β, pSer256-FOXO1A and BCL-2 levels and increases in P27 levels (**Figure 6D**). Therefore, the AKT pathway plays a key role in regulating the growth and Ara-C resistance of AML-M5 cells.

#### The FKBP51 Inhibitor Promotes the Growth of U937 Cells and Increases Resistance to Ara-C by Upregulating AKT Activity

**Figure 7:**
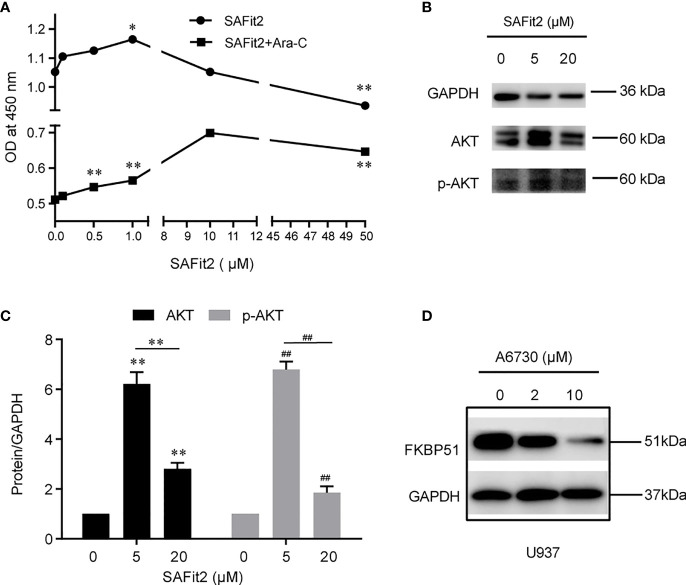
The inhibitor of FKBP51 promote the proliferation of AML-M5 cells and inhibit their sensitivity to Ara-C. **(A)** After treatment with different concentrations of SAFit2 for 48 hours with or without 0.05 μM Ara-C, U937 cell proliferation was assessed by CCK-8 assay. **(B, C)** U937 cells were treated with different concentrations of SAFit2 (0, 5, and 10 μM) for 48 hours and then stimulated with LPS (1 μg/ml) for 15 minutes. The protein expression levels of AKT and p-AKT were detected by western blot. GAPDH was the internal control. **(D)** Western blot analysis of FKBP51 in U937 cells after treatment with different concentrations of A6730 (0, 5 and 10 μM) for 48 hours. Statistical differences from negative control cells are indicated by asterisks (*p < 0.05, ** and ## for p < 0.01).

To confirm the above results, we used SAFit2 to inhibit the function of FKBP51 in U937 cells treated with or without Ara-C and measured cell viability and protein expression. The CCK-8 results showed that the OD450 increased gradually and peaked at SAFit2 1 µM (SAFit2 alone) and SAFit2 10 µM (SAFit2 in combination with 0.05 µM Ara-C and then decreased (**Figure 7A**). The western blot results indicated that pSer473-AKT and total AKT were increased at lower SAFit2 concentrations (5 µM) and decreased at higher SAFit2 concentrations (20 µM) (**Figures 7B, C**). These results were mostly consistent with those of the FKBP51-knockdown U937 cell experiments and implied that SAFit2 reverses the growth inhibition, Ara-C sensitivity, and hypophosphorylation of AKT in U937 cells induced by high FKBP51 expression. These findings verified that FKBP51 inhibits AML-M5 cell growth and Ara-C resistance through the AKT pathway.

#### The AKT Inhibitor Decreases the Expression of FKBP51

Do changes in AKT pathway activity in turn regulate the expression of FKBP51? We pretreated U937 cells with different concentrations of AI(0, 2 and 10 µM) for 48 hours and detected the FKBP51 protein. The western blot results indicated that the expression of FKBP51 was reduced in an AI concentration-dependent manner (**Figure 7D**). This suggested that the AKT pathway positively regulates the expression of FKBP51.

## Discussion

AML-M5 is an aggressive disease that requires new drug targets and therapeutic options. Strong expression of FKBP51 protein is observed in a variety of tissues and cell types, including kidney, liver, placenta, heart peripheral blood leukocytes (23). Recently, the role of FKBP51 in regulating tumorigenesis and tumor response to chemotherapy has been recognized in some cancers (13, 16, 22, 24). However, its role in human AML-M5 is not known. U937 cells originate from tissue and thus are more mature, whereas THP-1 cells originate from a leukemic blood and are thus less mature (25, 26). These two cell lines were selected to more objectively and accurately reflect the role of FKBP51 in AML-M5 cells. We knocked down FKBP51 in U937 cells and overexpressed it in THP-1 cells because the baseline expression of FKBP51 was high in U937 cells and low in THP-1 cells (**Figures 1A–D**).

In this study, we first proved that FKBP51 overexpression induces G0/G1 cell cycle arrest and inhibits cell proliferation in THP-1 cells both *in vitro* and *in vivo* (**Figures 2A, C, E–G**), and FKBP51 knockdown accelerates cell cycle progression and promotes cell proliferation in U937 cells compared to the control group (**Figures 2B, D**). These results suggested that FKBP51 negatively regulates AML-M5 cell growth. We also showed that the IC50 value of Ara-C, a first-line agent against AML (27), in FKBP51 overexpressed THP-1 cells was decreased while that in FKBP51-knockdown U937 cells was increased compared with the level in the control group (**Figures 4A, B, E, F**). In order to validate this observation, we injected FKBP51 overexpressed THP-1 cells and control cells into NOD/SCID mice to test their tumor formation ability and growth *in vivo*. Consistently, the tumor formation ability of the cells with FKBP51 protein overexpression was significantly weaker than that of the controls (**Figures 2E–G**). All of these results indicate that the expression of FKBP51 protein is involved in carcinogenesis and growth of AML-M5. Next, we analyzed if high or low FKBP51 expression altered cellular apoptosis of AML-M5 cells. Flow cytometry analyses showed that FKBP51 overexpression significantly promote Ara-C induced THP-1 apoptosis (23.8% and 16%, respectively, for THP-FKBP51 and THP-NC) while FKBP51-sh U937 cells showed apoptosis resistance. These data indicated that the expression level of FKBP51 is positively correlated with AML-M5 cells sensitivity to Ara-C. Our results are consistent with those of Furukawa Y et al, who reported that the IC80 value of Ara-C in THP-1 cells was higher (low FKBP51 expression, 720 nM) than that in U937 cells (high FKBP51 expression, 16 nM) (28).

FKBP51 was originally identified as a component of steroid receptor complexes (29). According to previous research, robust glucocorticoid-induced expression of FKBP51 has been observed in blood cells (30–32) and adipose tissue (33) and these studies indicated that FKBP51 may be a reliable and practical biomarker in predicting the response to corticosteroids in different diseases. However, here we want to explore the potential therapeutic effect of Dex in the treatment of AML-M5. We found increased FKBP51 expression in monocytic lines (THP-1 and U937) after addition of various concentrations of Dex to the cell culture medium (**Figures 3A–D**). We further tested the *in vitro* activity of dexamethasone as a single agent against AML cell lines. As expected, dexamethasone had slight activity in THP-1 and U937cell lines cultured in suspension (**Figures 3E, F**). The proliferation inhibition effect of Dex did not increase even the concentration increased more than tenfold. This indicated that THP-1 and U937 cells are refractory to growth inhibition by the dexamethasone. According to the fact that Dex can upregulate the expression of FKBP51 and FKBP51 enhance the sensitivity of AML-M5 cells to Ara-C, we further explored the effect of Dex on the chemosensitivity of Ara-C. We found Dex enhances the sensitivity of AML-M5 cells to Ara-C by up-regulating FKBP51. The IC50 decreased after Dex added, but this effect disappeared when FKBP51 knockdown (**Figures 4A–H**). Moreover, using a xenotransplantation model, we demonstrated that combination of Ara-C/Dex had a better anti-leukemic effect than Ara-C alone, while the Dex monotherapy also showed an anti-leukemic effect (**Figures 4J–L**). Here Ara-C(30mg/kg/d) is equivalent to the conventional doses in AML chemotherapy (34) and Dex 5mg/kg/d is equivalent to high therapeutic glucocorticoid dose in humans. Recently, several studies have explored the potential uses of steroids in the treatment of AML. The addition of dexamethasone to intensive chemotherapy results in a significant reduction in relapse and overall better survival rate in already hyperleucocytic AML patients (35). Addition of high-dose methylprednisolone to cytotoxic chemotherapy increased the remission rate and improved the outcome of the patients (27). Azoulay É et al. demonstrated that adding Dex to the chemotherapy regimen in AML-M5 patients with acute respiratory failure significantly diminished ICU mortality (12). The time may be come to extend glucocorticoid use to AML-M5 but future studies are needed to identify the best glucocorticoid for specific phases of treatment and the optimum duration and dose.

The PI3K/AKT pathway plays a critical role in preventing apoptosis and the pathogenesis of cancer. The constitutive activation of PI3K/AKT is associated with poor prognosis in AML (36, 37). FKBP51 is believed to serve as a scaffolding protein that recruits PHLPP to Akt to facilitate dephosphorylation in pancreatic cancer and endometrial adenocarcinomas (15, 21, 22).In this study, we found FKBP51 downregulated is related to Ara-C resistance in AML-M5. Hence the relationship between the expression level of FKBP51 and the activity of the AKT pathway in AML cells was investigated. As shown in **Figure 5A**, FKBP51 overexpression significantly decreased the pSer473 level of AKT in THP-1 cells, while its knockdown increased the pSer473 level of AKT in U937 cells. To identify the precise downstream effectors activated by AKT to regulate AML-M5 cell survival, we examined the expression of GSK3β, FOXO1A, BCL-2, BAX, and the cyclin-dependent kinase inhibitors (CDKIs) P27 and P21, which regulate cell survival and physiology (38–41). Our results indicated that FKBP51 overexpressed THP-1 cells showed reduced pSer9-GSK3β, pSer256-FOXO1A and BCL-2 and elevated P21 and P27. In contrast, FKBP51-knockdown U937 cells showed the opposite results. The proapoptotic protein BAX was unaffected by high or low FKBP51 levels (**Figures 5A, B**). Therefore, we speculated that FKBP51 overexpression induces dephosphorylation of AKT at Ser473, results in the accumulation of GSK3β and FOXO1A in the nucleus, and promotes gene transcription of P21 and P27, which prevent cell cycle progression in the G0/G1 phases by inhibiting cyclin D1 CDK4/6 complex formation (41–43). Meanwhile, reduced BCL-2 attenuates the inhibition of apoptosis. Ultimately, FKBP51 overexpression inhibited the growth of AML-M5 cells, while FKBP51 knockdown led to the opposite result. Further, we measured cell growth indicators after treating the cells with AI. The viability of all groups of cells decreased in an AI concentration-dependent manner (AI 1.8, 5.4 and 9 μM) (**Figure 6A**) as well as decreased cell cycle arrest (AI 5.4 µM) (**Figures 6B, C**). These results emphasized that FKBP51 negatively regulates the activity of the AKT pathway and thus inhibits AML-M5 cell growth.

To further verify the role of FKBP51 in the growth of AML-M5 cells, we used SAFit2, a highly selective FKBP51 ligand (44), to pre-treat U937 cells with or without Ara-C (0.05 μM) and performed a CCK-8 assay. As shown in **Figure 7A**, cell viability increased gradually and peaked at 1 μM SAFit2 (without Ara-C) and 10 μM SAFit2 (with 0.05 μM Ara-C). Accordingly, pSer473-AKT and total AKT increased after SAFit2 treatment (**Figures 7B, C**). These results demonstrated that SAFit2 reverses FKBP51-induced cell growth inhibition and Ara-C sensitivity by abolishing the dephosphorylation of Ser473 of AKT by FKBP51. These results were consistent with those in FKBP51-knockdown U937 cells. In summary, our study demonstrated that FKBP51 inhibits AML-M5 cell growth and reduces cell resistance to Ara-C by inhibiting the activity of the AKT pathway.

Several studies have shown that the AKT pathway is required for the expression of some genes in monocytes/macrophages and leukemia cells (45–47). To determine whether the activity of the AKT signaling pathway influences FKBP51 expression in AML-M5 cells, we assessed the expression of FKBP51 protein after inhibition of AKT activation using AI in U937 cells. As shown in **Figure 7D**, the expression of FKBP51 significantly decreased in an AI concentration-dependent manner. This result suggested that the AKT pathway positively regulates the expression of FKBP51 in AML-M5 cells. Given our results above, we hypothesized that the expression of FKBP51 and the activity of the AKT pathway in AML-M5 cells are mutually regulated. Low expression of FKBP51 promotes phosphorylation at Ser473 and activates the AKT pathway, which in turn increases the expression of FKBP51. Elevated FKBP51 expression dephosphorylates Ser473 and inactivates the AKT pathway. This cycle is repeated to form a feedback loop, which may help to maintain the activity level of the AKT pathway. Interestingly, these results may explain why high concentrations of SAFit2 failed to maintain high levels of cell proliferation and the expression of pSer473 and total AKT (**Figures 7A–C**). Of course, both AKT and FKBP51 are intricately regulated by a number of other factors. The mechanisms and pathways involved need further study.

In summary, we believe that the results obtained in experimental studies both in mice and in THP-1 and U937 cells that Dex enhanced Ara-C sensitivity by upregulating FKBP51 will provide important benefits for the outcome of patients with AML-M5 and possibly for patients with other malignancies. Rationally designed combination approaches may be more effective than single-agent, and may be the approach to pursue in AML. However, this therapeutic strategy should be explored in a clinical trial of AML-M5 patients in the future.

## Data Availability Statement

The raw data supporting the conclusions of this article will be made available by the authors, without undue reservation.

## Ethics Statement

The animal study was reviewed and approved by Medical Ethics Committee of Shandong Provincial Hospital.

## Author Contributions

YZ, YJ, and HS conceived and designed the study. HS, XWL, LW, BC, WM, YX, SL, and XL performed the experiments. HS, YJ, and XWL analyzed the data and performed the statistical analyses. HS, YJ, and YZ wrote the paper. All authors contributed to the article and approved the submitted version.

## Conflict of Interest

The authors declare that the research was conducted in the absence of any commercial or financial relationships that could be construed as a potential conflict of interest.

## Publisher’s Note

All claims expressed in this article are solely those of the authors and do not necessarily represent those of their affiliated organizations, or those of the publisher, the editors and the reviewers. Any product that may be evaluated in this article, or claim that may be made by its manufacturer, is not guaranteed or endorsed by the publisher.
